# Bioelectrical impedance analysis versus quantitative computer tomography and anthropometry for the assessment of body composition parameters in China

**DOI:** 10.1038/s41598-021-90641-5

**Published:** 2021-05-26

**Authors:** Qian Qin, Yang Yang, Jingfeng Chen, Yaojun Jiang, Ang Li, Meng Huang, Yihan Dong, Shoujun Wang, Suying Ding

**Affiliations:** 1 Health Management Center, The First Affiliated Hospital of Zhengzhou University, Jianshe Road, Zhengzhou, China; 2 Radiology Department, The First Affiliated Hospital of Zhengzhou University, Jianshe Road, Zhengzhou, China; 3 Endocrinology Department, The First Affiliated Hospital of Zhengzhou University, Jianshe Road, Zhengzhou, China

**Keywords:** Endocrinology, Health care

## Abstract

Obesity, especially abdominal obesity, is correlated to increased risk of cardiovascular morbidity and mortality. It is urgent to search a simply method to predict visceral fat area (VFA). Herein, we evaluated the correlation of waist circumference (WC) measured by anthropometry and bioelectrical impedance analysis (BIA), and VFA estimated by BIA or measured by quantitative computed tomography (QCT) in China. The mean body mass index (BMI) was 25.09 ± 3.31 kg/m^2^ and the mean age was 49.16 ± 9.19 years in 2754 subjects. VFA-BIA were significantly smaller than VFA-QCT in both BMI and age subgroups between male and female (*p* < 0.001). High correlation was observed for WC between BIA and manually (*r* = 0.874 for all, *r* = 0.865 for male and *r* = 0.806 for female) and for VFA between BIA and QCT (*r* = 0.512 for all). The intraclass correlation coefficient (ICC) showed the perfect agreement between BIA and manually to measure WC (ICC = 0.832 for all, 0.845 for male and 0.697 for female) and implied a good reliability for VFA between BIA and QCT with women among subgroups (ICC = 0.623 for all, ICC = 0.634 for age < 50 years and ICC = 0.432 for BMI > 24 kg/m^2^), whereas the good reliability was lost in men (ICC = 0.174). The kappa analysis showed a moderate consistency for VFA measured by BIA and QCT (Kappa = 0.522 with age < 50 years, 0.565 with age ≥ 50 years in male; Kappa = 0.472 with age < 50 years, 0.486 with age ≥ 50 years in female). In addition, BIA to estimate VFA (*r* = 0.758 in male, *r* = 0.727 in female, *P* < 0.001) has a stronger correlation with VFA measured by QCT than BMI and WC according to gender categories. Furthermore, ROC analysis showed the cut-off point of VFA measured by BIA for predicting visceral obesity was: 101.90 cm^2^, 119.96 cm^2^ and 118.83 cm^2^ and the Youden’s index was 0.577, 0.577 and 0.651, respectively and the Kappa value was 0.532, 0.536 and 0.611 in unadjusted model, model 1 and model 2. In conclusion, being non-invasive and free of radiation, BIA can be used as a safe and convenient tool to estimate VFA in female; especially for monitoring the VFA of the same person, the BIA has superiority to a certain extent. However, the consistency is not most ideal between BIA and QCT. When using BIA to assess whether a person is visceral obesity, we must take into consideration age, BMI and WC. Therefore, we established a regression formula to reflect VFA-QCT by VFA-BIA, age, BMI, and WC. In addition, a more accurate formula is needed to match the CT data in China.

## Introduction

Obesity is an independent risk factor for diseases, such as hypertension, diabetes, hyperlipidemia, cardiovascular and cerebrovascular diseases^1–3^, which brings tremendous economic burden, and has become a leading public health challenge globally. The World Health Organization (WHO) defines obesity as abnormal or excessive fat accumulation that may cause health damage^4^. In recent years, studies have shown that in addition to the fat area, especially visceral fat, these have an inseparable relationship with insulin resistance^5,6^.

At present, body composition parameters were measured manually or using bioelectrical impedance analysis (BIA) and quantitative computer tomography (QCT). Previous researchers have often used body mass index (BMI), waist circumference (WC), hip circumference (HC) and waist-hip ratio (W/H) by manual measurement as criteria to define obesity. However, these variables could not differentiate subcutaneous and visceral fat. In recent years, QCT has been used to assess intra-abdominal obesity according to the intra-abdominal fat level of > 100 (cm^2^)^7^. QCT can accurately distinguish between subcutaneous and visceral fat, and be unaffected by the abdominal contents. This has been considered an accurate method to measure overall adiposity and fat distribution^8,9^. QCT has radiation. Therefore, there is concern on the use of QCT for the safety of the people. In addition, QCT is expensive. Thus, this may have restrictions on its availability for some patients due to financial concerns. In comparison, BIA is free of radiation, non-invasive and easy-to-use. This is also cheaper than QCT. In addition, visceral fat area (VFA) was also estimated by a multifrequency BIA^10–12^. Therefore, BIA has been widely used in clinics and fitness centers for the monitoring of overall adiposity and fat distribution. However, there is scarce evidence regarding the accuracy of BIA against that of manual measurements and QCT. Therefore, it is of utmost importance to choose a safe device with high accuracy and sensitivity to measure overall adiposity and fat distribution, in order to evaluate obesity and predict obesity-related diseases.

Therefore, the present study validated BIA against manual measurement and QCT to evaluate the reliability and accuracy of BIA. The present study was conducted on 2754 Chinese subjects with a wide age range (20–81 years old), aiming to explore the best method for measuring body composition parameters.

## Materials and methods

### Research subjects

The present study was part of the China Biobank project, which is a prospective nationwide multicenter cohort study. The subjects were recruited from the First Affiliated Hospital of Zhengzhou University, which is the study center of the multi-center China Biobank cohort. This study was conducted in keeping with the Helsinki Declaration and Rules of Good Clinical Practice. The study was approved by the Institutional Review Board of the First Affiliated Hospital of Zhengzhou University (2018-KY-56). Written informed consents were signed by all participants at the time of registration. These subjects were recruited during their physical examination in the Hospital between January 2018 and December 2020. Subjects within the age of 20–81 years old were included. Subjects were excluded according to the following criteria: (1) females who were pregnant or planning for pregnancy; (2) patients with metal implants during the upper abdomen scan; (3) patients with severe cardiopulmonary diseases, other serious systemic diseases, malignant tumors, and severe abdominal or metabolic diseases that could affect the distribution of abdominal fat; (4) patients under glucocorticoid treatment. A total of 2,754 subjects were recruited for the present study. After overnight fasting of food and water, these subjects underwent BIA, QCT and manual measurements with an empty stomach and bladder. All subjects provided a written informed consent, and the study was approved by the Ethics Committee of the First Affiliated Hospital of Zhengzhou University. The present study has also been registered with the US clinical trials database (https://clinicaltrials.gov/ct2/show/NCT03699228; trial identifier: NCT03699228 (01/12/2017); China Nationwide Multi Center Big Data Study on the Quantitative Computed Tomography (QCT) and Health Status of Check-Up Population). The present study used the baseline data of all recruited subjects.

### Physical parameter measurement

Anthropometric data (body weight, height and WC) were determined using an integrated standard method with replicate measurements for two times. Height was measured with the principle of ultrasonic wave with SK-X80 (Sonka Shenzhen China). Height measuring instrument is through the continuous ultrasonic reflection echo after launch obstacles to measure the time lag between the transmitting and receiving echoes, and then through the wave speed multiplied by time to calculate the distance. Waist circumference was measured in standing position at the midpoint between the lateral iliac crest and the lowest rib. BMI was calculated as weight (kg) divided by height squared (m^2^). These subjects were categorized as underweight, normal weight, overweight and obese, according to the definition of obesity of the WHO for the Asia–Pacific region: underweight BMI < 18.5 (kg/m^2^), normal 18.5–23.9 (kg/m^2^), overweight 24–27.9 (kg/m^2^), and obese BMI ≥ 28 (kg/m^2^)^13^. VFA and WC was estimated by a multifrequency bioelectrical impedance analysis (BIA) device (InBody 770, InBody Co., Ltd., Korea) with tetrapolar electrodes^10–12,14,15^. The participants were requested to forbid eating, drinking and strenuous exercise for 4 h prior to measurement. Subject age, sex and height data were entered into the BIA machine. After confirming that the subject was standing correctly with both arms apart from the body, both feet on the right spots on the platform and with minimum clothing and without shoes, a supervisor pushed the start button to perform assessment. Both hands were held at a 45 degree angle away from the body. X-scan uses 1 kHz, 5 kHz, 50 kHz, 250 kHz, 550 kHz, and 1000 kHz frequencies to analyze intracellular and extracellular fluid values and water. All BIA measurements were performed by the same investigator.

### Scanning and measurement of abdominal QCT

CT scans at the L2-L3 level was performed with the Brilliance iCT Elite FHD device to measure the visceral fat area (VFA) underling scan parameters (120 kV, 41 mAs for weight > 70 kg, 19 mAs for weight ≤ 70 kg, 5-mm slice thickness and spacing for scanning, 1-mm slice thickness and spacing for reorganization, 0.5 s rotation time, 512^2^ pixel matrix, and 500-mm display field of view and bed height based on the midaxillary line). The cross-sectional abdominal contour was estimated by manually delineating the skin with a graph pen through the muscular structures and vertebral corpora. The area was automatically calculated using dedicated software (QCT PRO V6.1, Mindways, USA). The data was reconstructed by iterative model reconstruction (IMR, level 2). All CT examinations were performed by two experienced radiologists.

### Statistical analysis

Continuous variables are reported as mean ± standard deviation (SD). The Spearman relation, Bland–Altman, ICC and Kappa analysis were used to calculate the correlation or reliability or consistency of prediction of WC and VFA measured by three methods. Multiple linear regression models were used to estimate VFA from BIA for against VFA values by QCT. According to the diagnostic criteria for visceral obesity (VFA ≥ 100 cm^2^ measured by QCT), Receiver Operating Characteristic Curve (ROC) was plotted to determine the cut-off point value to predict visceral obesity by BIA. The formula was considered the following factors: gender, age, BMI, and VFA-BIA. The following cutoff values were used to interpret Spearman correlations: *r* < 0.20 = very weak; 0.20 to 0.39 = weak; 0.40 to 0.59 = moderate; 0.60 to 0.79 = strong; and 0.80 to 1.0 = very strong^16^. The cutoff values to interpret the ICC were as follows: < 0.20 = slight; 0.20 to 0.39 = fair; 0.40 to 0.59 = moderate; 0.60 to 0.79 = substantial; and 0.80 to 1.0 = almost perfect^17^. The cutoff values to interpret the weighted kappa were as follows: < 0.20 = poor; 0.20 to 0.40 = fair; 0.41 to 0.60 = moderate; 0.61 to 0.80 = good; and 0.81 to 1.0 = very good^18^. The statistical analyses were performed using the R software (version 3.6.1)^19^ and MedCalc statistical software (version 19.7.2). R software was used to compute the Pearson’s correlation coefficient using “cor” function and draw association plot using “ggplot2” package. Bland–Altman plot was drawn using “BlandAltmanLeh” package. Multivariate regression model with tenfold cross-validation method was performed using “createFolds” function in “caret” package and “lm” function, and then selected the optimal regression equation based on the minimum Mean Square Error (MSE). Calibration plot was drawn using “rms” package. Statistical significance was defined as a two-sided *P*-value of ≤ 0.05. MedCalc statistical software was used to compute the cut-off points and weighted kappa values, and drew the ROC.

## Results

### Subject characteristics

After strict exclusion standard, we chose 2754 subjects from January 2018 to December 2020 ultimately. However, WC of 1436 subjects was measured by anthropometry. The detailed male and female characteristics are presented in Table 1. The mean ± SD of WC were 89.46 ± 10 cm and 89.31 ± 10.30 cm by BIA and manually, respectively. Men had a greater mean WC than women by about 8.66 cm and 11.77 cm by BIA and manually. Compared to males, females had lower VFA by QCT; whereas, when compared to the method of BIA, females had lower VFA by QCT (*p* < 0.001).

**Table 1:** The baseline characteristics of the study population.

	All (2754)	Male (1491)	Female (1263)	*t* (*z*)	*P*
Age (year)	49.6 ± 9.19	49 ± 9.57	50.3 ± 8.68	3.70	< 0.001
Height (cm)	165.84 ± 8.13	171.18 ± 6.04	159.53 ± 5.25	− 53.49	< 0.001
Weight (kg)	69.32 ± 12.41	76.3 ± 11	61.08 ± 8.21	− 40.53	< 0.001
BMI (Kg/m^2^)	25.09 ± 3.31	26 ± 3.19	24.01 ± 3.11	− 16.48	< 0.001
WC-BIA (cm) (BIA)	89.46 ± 10	93.43 ± 9.64	84.77 ± 8.23	− 25.10	< 0.001
WC-Anthropometry (cm)	89.31 ± 10.30	91.94 ± 8.51	80.17 ± 8.40	− 26.35	< 0.001
VFA-BIA (cm^2^)	100.68 ± 34.28	96.52 ± 32.57	105.59 ± 35.59	6.98	< 0.001
VFA-QCT (cm^2^)	174.18 ± 79.81	216.85 ± 73.84	123.81 ± 52.59	− 37.45	< 0.001

### The correlations of WC between manual measurement and BIA

We analyzed the correlations of WC of 1436 subjects between anthropometry and BIA. The method of BIA to estimate WC was significantly higher than manual measurements (*P* < 0.001), The Spearman *r* showed a strong correlation with WC between BIA and manually in Table 2. The ICC also demonstrated perfect agreement between two methods in Table 2. The bias (BIA-manual) was positive overall (4.56), for males (1.84) and for females (4.55) with Bland–Altman analysis, which implied the BIA method overestimated WC compared with the method of anthropometry. We found that a higher consistent in males than in females between two measurements. A good reliability was found between the WC measured by BIA and the manual method because the percentage error was less than 15% overall (11.63%), for males (6.0%) and for females (12.51%).

**Table 2:** Correlation coefficient (*r*), ICC, and Bland–Altman analysis between anthropometry and BIA for WC.

	Total (1436)	Male (749)	Female (687)
Pearson *r*	0.874***	0.865***	0.806***
ICC	0.832	0.845	0.697
**Bland–Altman analysis**			
Bias (BIA-manual)	4.56	1.84	4.55
SD	5.12	4.67	5.12
95% LOA	− 5.48 to 14.59	− 7.31 to 10.99	− 5.49 to 14.59
Percentage error (%)	11.63%	6.0%	12.51%

### The correlation of VFA between QCT and BIA

**Figure 1:**
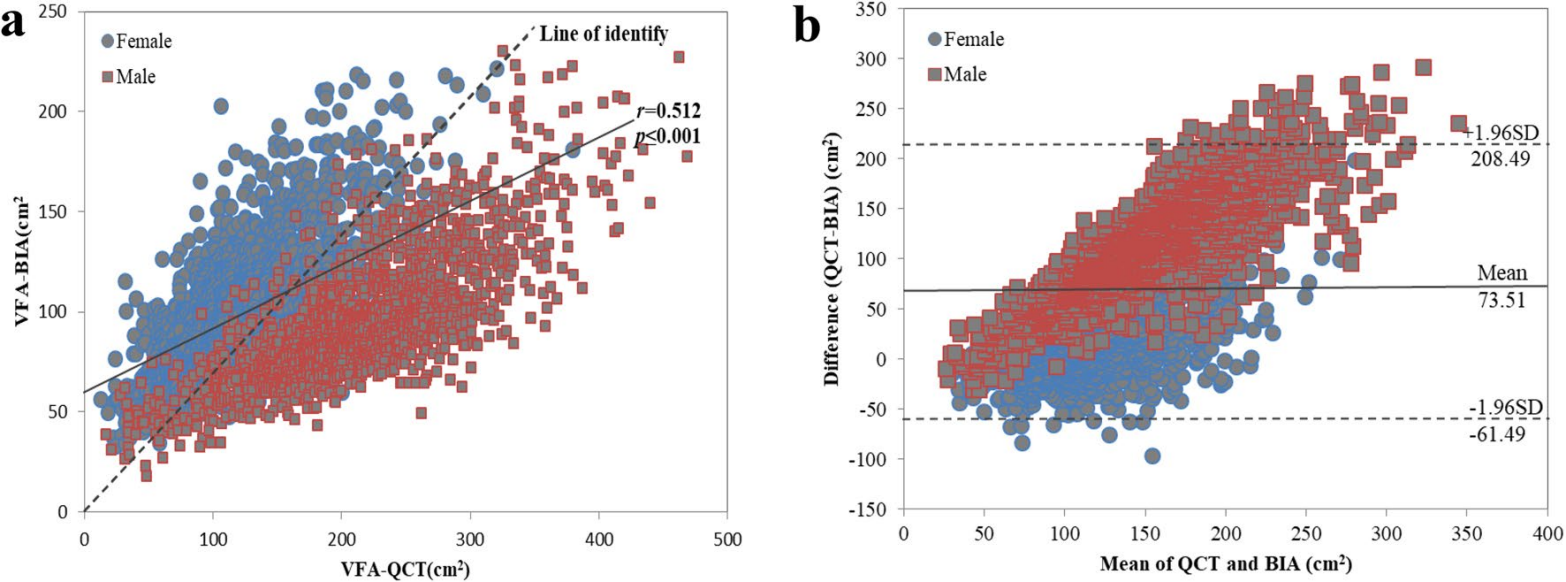
The association and consistency of VFA measured by QCT and BIA. (**a**) Scatter plot of VFA measured by QCT and BIA. VFA estimated by BIA had significantly positive correlation with that of the QCT method (*r* = 0.512, *P* < 0.001). (**b**) Bland–Altman plot of difference in VFA (QCT measurement minus BIA measurement) against the mean of two measurements. The middle line denotes bias (mean difference between the two measurements), and dashed lines denote 95% limits of agreement (1.96 SD of the difference, bias: 73.51, 95% LOA: − 61.49 to 208.49).

The VFA-BIA was positively correlated with the VFA-QCT (*r* = 0.512, *p* < 0.001), according to the Pearson’s correlation test (Fig. 1a). The Bland–Altman analysis showed that VFA of bias (QCT-BIA) was positive overall (73.51), for males (120.34) and for females (18.22) (Table 3 and Fig. 1b), indicating an underestimation of the BIA compared with VFA-QCT (> 100 cm^2^) and an overestimation VFA in those having a lower VFA-QCT (< 100 cm^2^).

**Table 3:** The consistency of the VFA between BIA and QCT using the Bland–Altman and ICC method according to the gender, BMI and age groups.

	N	VFA-QCT (cm^2^)	VFA-BIA (cm^2^)	VFA-QCT-VFA-BIA (cm^2^)	*p	^†^*p*	ICC
Total	2754	174.18 ± 79.81	100.68 ± 34.28	73.51 ± 68.87	< 0.001		0.216
Gender						< 0.001	
Male	1491	216.85 ± 73.84	96.517 ± 32.57	120.34 ± 53.53	< 0.001		0.174
Female	1263	123.81 ± 52.59	105.59 ± 35.59	18.22 ± 36.23	< 0.001		0.623
BMI (kg/m^2^)						< 0.001	
< 18.5	33	49.66 ± 22.04	46.37 ± 11.63	3.29 ± 19.17	0.332		0.408
18.5–23.9	1024	116.31 ± 51.18	77.64 ± 20.29	38.67 ± 50.56	< 0.001		0.105
24–27.9	1210	192.83 ± 60.61	104.19 ± 24.61	88.64 ± 65.44	< 0.001		0.001
28–29.9	262	237.04 ± 63.55	128.3 ± 26.47	108.74 ± 72.42	< 0.001		− 0.031
≥ 30	225	282.33 ± 76.63	162.42 ± 28.42	119.91 ± 78.81	< 0.001		0.022
Age (years)						< 0.001	
20–39	347	161.48 ± 82.84	97.37 ± 36.97	64.11 ± 66.07	< 0.001		0.313
40–49	1036	164.05 ± 81	95.35 ± 30.43	68.7 ± 70.9	< 0.001		0.202
50–59	1016	182.66 ± 76.95	104.98 ± 35.41	77.68 ± 68.31	< 0.001		0.190
60–69	295	188.38 ± 74.08	104.74 ± 34.05	83.65 ± 64.92	< 0.001		0.178
≥ 70	60	209.27 ± 79.96	118.96 ± 43.91	90.31 ± 64.89	< 0.001		0.251
**Male (1491)**							
BMI (kg/m^2^)						< 0.001	
< 24	378	147.21 ± 54.79	65.96 ± 15.52	81.25 ± 46.15	< 0.001		0.113
≥ 24	1113	240.51 ± 63.88	106.89 ± 30.24	133.61 ± 49.22	< 0.001		0.113
Age (Years)						< 0.001	
< 50	774	211.32 ± 71.64	96.25 ± 32.65	115.07 ± 52.38	< 0.001		0.178
≥ 50	717	222.82 ± 75.75	96.8 ± 32.5	126.02 ± 54.22	< 0.001		0.170
**Female (1263)**							
BMI (kg/m^2^)						< 0.001	
< 24	679	95.87 ± 39.65	82.62 ± 20.98	13.25 ± 33.05	< 0.001		0.421
≥ 24	584	156.29 ± 46.82	132.3 ± 30.01	24 ± 38.83	< 0.001		0.432
Age (years)						< 0.001	
< 50	609	102.5 ± 43.73	95.36 ± 31.62	7.15 ± 32.14	< 0.001		0.634
≥ 50	654	143.65 ± 52.4	115.12 ± 36.45	28.53 ± 36.79	< 0.001		0.557

### Subgroups comparison between VFAs by QCT and BIA according to gender, BMI, and age by ICC analysis

To analysis the concordance between VFA-BIA and VFA-QCT, we divided all subjects into different subgroups according to gender, BMI (< 18.5, 18.5–23.9, 24–27.9, 28–29.9, ≥ 30 kg/m^2^) and age (20–39, 40–49, 50–59, 60–69 and ≥ 70 years).

The VFA-QCT was higher than the VFA-BIA among all subjects, genders, BMI and age subgroups (Table 3). The mean difference was larger in male than female (120.34 ± 53.53 vs. 18.22 ± 36.23 cm^2^, *p* < 0.001). The ICC value in female was 0.623 between the two methods suggesting good reliability. Among the BMI categories, the ICC (ICC = 0.408) in BMI < 18.5value was higher than other BMI subgroups. Among the age subgroups, the ICC of age in 20–39, 40–49 and ≥ 70 years was 0.313, 0.202 and 0.251, respectively and the ICC value were less than 0.2 in other BMI and age subgroups (Table 3).

To determine further whether the concordance of VFA between QCT and BIA differed in gender, we divided all subjects into two subcategories: lean and normal (BMI < 24 kg/m^2^) vs. overweight and obese (BMI ≥ 24 kg/m^2^); and younger (age < 50 years) vs. older (age ≥ 50 years) according to gender. VFA-BIA were significantly smaller than VFA-QCT both in BMI and age subgroups between male and female (p < 0.001, Table 3). VFA-BIA in female with BMI < 24 kg/m^2^ or age < 50 years were similar to VFA-QCT (mean differences were 13.25 ± 33.05 cm^2^ in the BMI < 24 kg/m^2^ group and 7.15 ± 32.14 cm^2^ in the age < 50 years group, Table3). The highest ICC value was 0.634 in female with age < 50 years (Table 3), which implied a similar tendency to the mean differences about VFA between two methods in female with age < 50 years; whereas the trend was missing among male.

### Subgroups comparison between VFAs by QCT and BIA according to gender and age by Kappa analysis

In addition, we analysis the concordance of VFA distribution by Kappa analysis. The VFA measured by QCT and BIA was used as the observation index and divided into four groups according to the quartile. We found that the overall consistency of the two methods is poor (Kappa = 0.295). However, there is moderate consistency between BIA and QCT in different subgroups according to gender and age. The moderate Kappa value was 0.522 with age < 50 years, 0.565 with age ≥ 50 years and 0.540 for whole age in male. The fair Kappa value was 0.472 with age < 50 years, 0.486 with age ≥ 50 years and 0.524 for whole age in female (Table 4).

**Table 4:** The consistency of the VFA between BIA and QCT using the Kappa method according to the gender and age groups.

Total (2754)	QCT-BIA (cm^2^)				Kappa
VFA-BIA (cm^2^)	Q1 (≤ 110.5)	Q2 (110.5 ~ 168.1)	Q3 (168.1 ~ 227.9)	Q4 (≥ 227.9)	
Q1 (≤ 75.7)	361	167	122	39	0.295
Q2 (75.7 ~ 95.6)	178	167	177	166	
Q3 (95.6 ~ 120.23)	124	220	171	174	
Q4 (≥ 120.23)	27	138	214	309	
Male (1491)	Q1 (≤ 169.9)	Q2 (169.9 ~ 213.6)	Q3 (213.6 ~ 264.0)	Q4 (≥ 264.0)	
Q1 (≤ 74.4)	254	88	27	7	0.540
Q2 (74.4 ~ 91.3)	88	149	99	35	
Q3 (91.3 ~ 112.2)	23	109	132	93	
Q4 (≥ 112.2)	8	27	115	237	
Male (774), Age < 50	Q1 (≤ 163.8)	Q2 (163.8 ~ 208.1)	Q3 (208.1 ~ 259.8)	Q4 (≥ 259.8)	
Q1 (≤ 73.9)	125	48	16	5	0.522
Q2 (73.9 ~ 90.6)	51	74	49	20	
Q3 (90.6 ~ 112.3)	13	58	75	48	
Q4 (≥ 112.3)	4	15	53	120	
Male (717), Age ≥ 50	Q1 (≤ 175.1)	Q2 (175.1 ~ 219.4)	Q3 (219.4 ~ 271.6)	Q4 (≥ 271.6)	
Q1 (≤ 75.1)	128	41	7	3	0.565
Q2 (75.1 ~ 92.3)	36	74	53	17	
Q3 (92.3 ~ 112.0)	12	57	66	44	
Q4 (≥ 112.0)	3	8	53	115	
Female (1263)	Q1 (≤ 85.3)	Q2 (85.3 ~ 117.2)	Q3 (117.2 ~ 156.7)	Q4 (≥ 156.7)	
Q1 (≤ 78.1)	203	81	21	11	0.524
Q2 (78.1 ~ 101.5)	95	107	93	21	
Q3 (101.5 ~ 126.9)	17	98	117	85	
Q4 (≥ 126.9)	2	29	85	198	
Female (609), Age < 50	Q1 (≤ 71.8)	Q2 (71.84 ~ 96.4)	Q3 (96.4 ~ 125.4)	Q4 (≥ 125.4)	
Q1 (≤ 70.9)	92	40	18	4	0.472
Q2 (70.9 ~ 89.5)	37	54	50	10	
Q3 (89.5 ~ 114.1)	20	42	46	44	
Q4 (≥ 114.1)	3	17	38	94	
Female (654), Age ≥ 50	Q1 (≤ 106.9)	Q2 (106.9 ~ 141.3)	Q3 (141.3 ~ 175.5)	Q4 (≥ 175.5)	
Q1 (≤ 90.1)	103	37	18	7	0.486
Q2 (90.1 ~ 113.6)	44	62	49	9	
Q3 (113.6 ~ 138.2)	13	48	50	51	
Q4 (≥ 138.2)	3	17	47	96	

### The correlations among VFA, WC and BMI between QCT and BIA

Among all participants, the VFA-QCT has no correlation with age. The method of BIA to estimate VFA (*r* = 0.758 in male, *r* = 0.727 in female, *P* < 0.001) has a higher correlation with VFA measured by QCT than BMI and WC according to gender categories in Table 5. The correlation is stronger in male than female among BMI (total, < 24 and ≥ 24 kg/m^2^), WC and VFA-BIA (Table 5).

**Table 5:** Correlation of age, BMI, WC and VFA by BIA with VFA by CT values.

	All (2754)		Male (1491)		Female (1263)	
	*r*	*p*	*r*	*p*	*r*	*p*
Age (years)	0.140	< 0.001	0.107	< 0.001	0.441	< 0.001
< 50	0.037	0.168	0.172	< 0.001	0.214	< 0.001
≥ 50	0.075	0.006	− 0.022	0.560	0.253	< 0.001
BMI (kg/m^2^)	0.720	< 0.001	0.721	< 0.001	0.697	< 0.001
< 24	0.509	< 0.001	0.534	< 0.001	0.491	< 0.001
≥ 24	0.520	< 0.001	0.580	< 0.001	0.486	< 0.001
WC-BIA (cm)	0.791	< 0.001	0.756	< 0.001	0.706	< 0.001
VFA-BIA (cm^2^)	0.512	< 0.001	0.758	< 0.001	0.727	< 0.001

### New formula to predict VFA-QCT using VFA-BIA data

**Figure 2:**
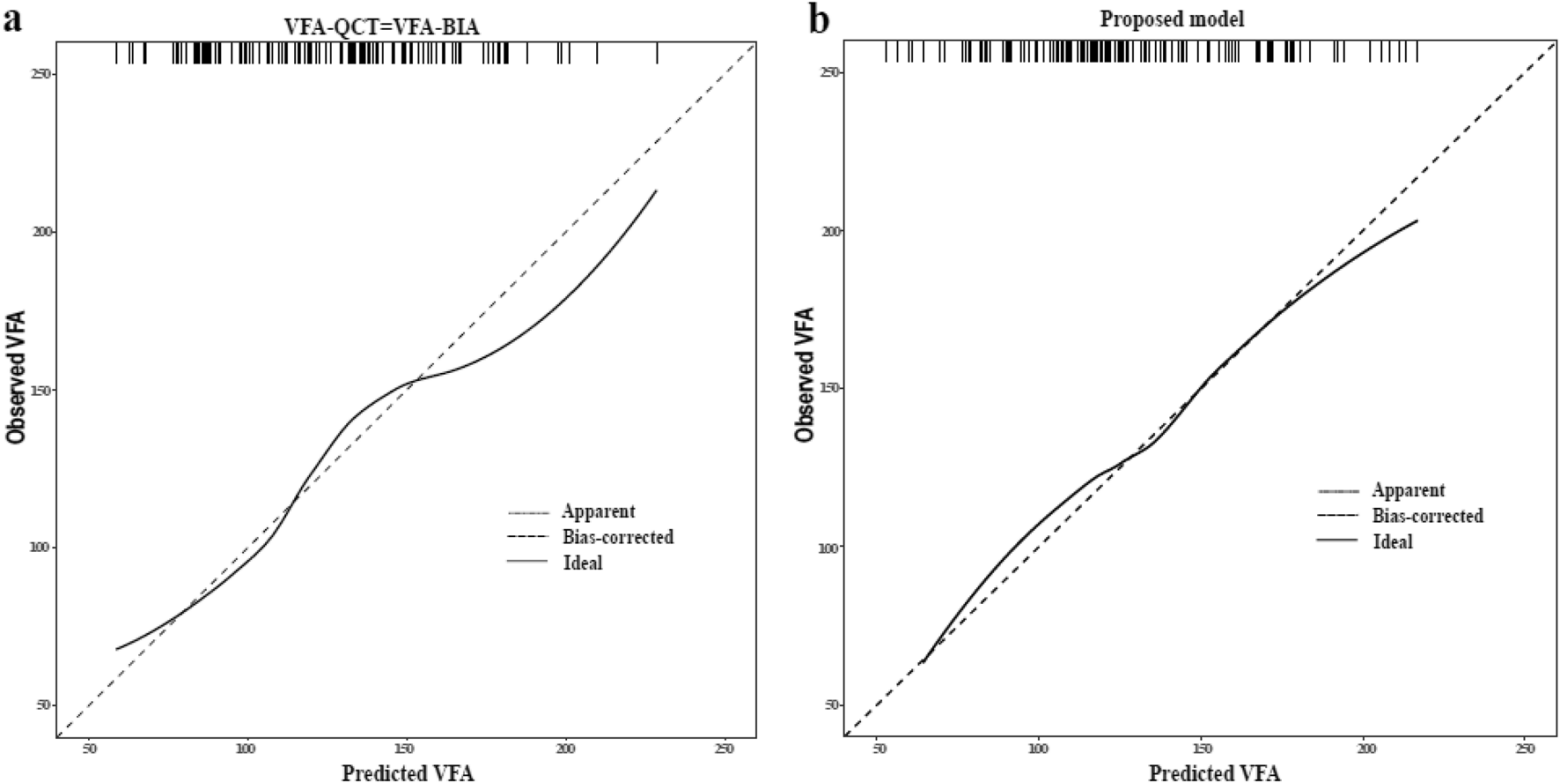
Calibration plots of prediction model in female. (**a**) A univariate regression analysis of predicted VFA by VFA-BIA (MSE = 1085.9). (**b**) A multivariate regression analysis of predicted VFA with VFA-BIA, age, BMI, and WC (MSE = 806.1).

After a minimum MSE selection with tenfold cross-validation method, the results of multiple linear regression were retained in the final models in female: VFA-BIA, age, BMI, and WC. Compared with a univariate prediction model with VFA-BIA only, the MSE decreased from 1085.9 to 860.1 in female, resulting in an improvement in the agreement between observations and predictions (Table 6 and Fig. 2).

**Table 6:** The Multivariate regression models estimate the VFA in females between BIA and QCT.

	*β*	S.D	*t*	*β* (95%CI)	*p*	MSE
VFA-BIA (only)						1085.9
Intercept	9.91	3.37	2.94	4.19 ~ 16.69	< 0.01	
VFA-BIA	1.08	0.03	35.8	1.01 ~ 1.13	< 0.001	
VFA-BIA, Age, BMI, and WC						806.1
Intercept	− 219.29	18.98	− 11.55	− 256.53 to − 182.05	< 0.001	
VFA-BIA	0.39	0.07	5.78	0.26 ~ 0.52	< 0.001	
Age	1.73	0.12	14.60	1.50 ~ 1.96	< 0.001	
BMI	2.90	0.76	3.82	1.41 ~ 4.39	< 0.001	
WC	1.71	0.33	5.16	1.06 ~ 2.36	< 0.001

The final models were derived as: y = (− 219.29) + 0.39*VFA-BIA + 1.73*Age + 2.90*BMI + 1.71*WC for female (Table 6).

### The cut-off point of VFA measured by BIA was predicted by ROC curve analysis for female

**Figure 3:**
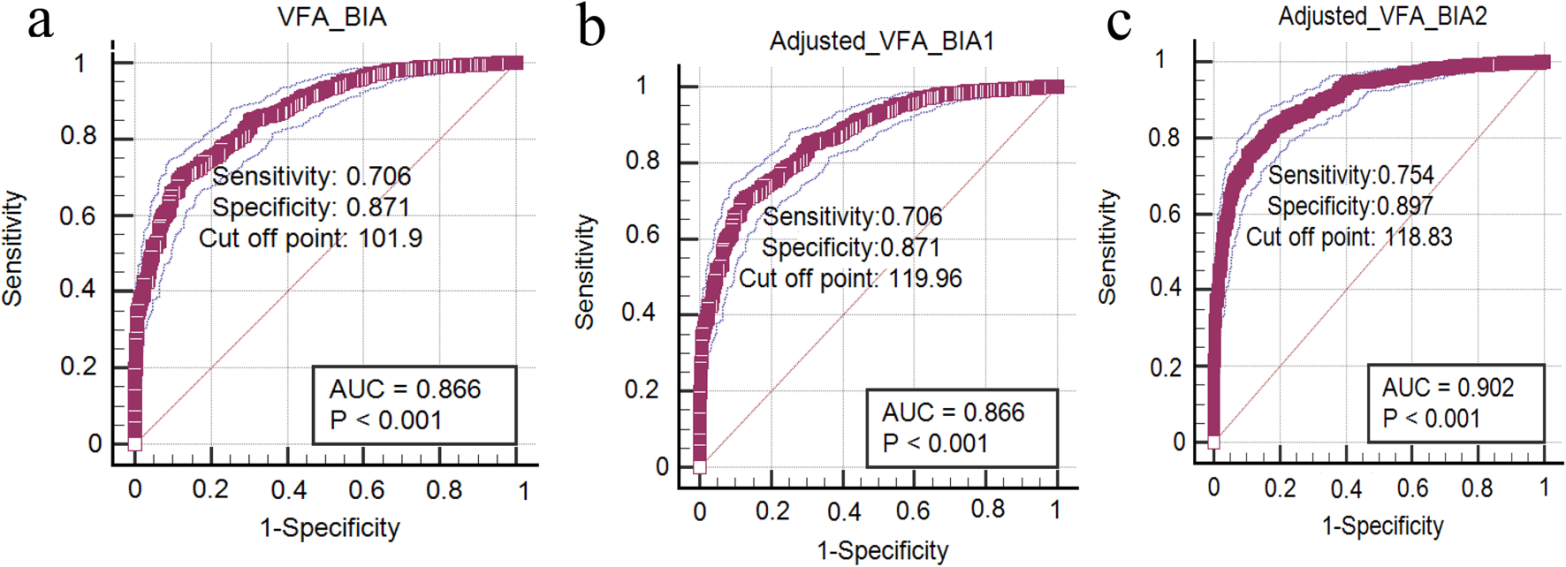
ROC of visceral obesity predicted by BIA in female. (**a**) The cut off point is 101.90 cm^2^ in unadjusted model. (**b**)The cut off point is 119.96 cm^2^ in model 1, which is the univariate regression analysis of predicted VFA-QCT by VFA-BIA. (**c**)The cut off point is 118.83 cm^2^ in model 2, which is the multivariate regression analysis of predicted VFA-QCT with VFA-BIA, age, BMI, and WC.

According to the diagnostic criteria for visceral obesity (VFA ≥ 100 cm^2^ measured by QCT), ROC analysis showed the cut-off point of VFA measured by BIA in unadjusted model, model 1 and model 2 to predict visceral obesity was: 101.90 cm^2^, 119.96 cm^2^ and 118.83 cm^2^ for female; the sensitivity was 0.706, 0.706, and 0.754 respectively; the specificity was 0.871, 0.871, and 0.897, respectively and the Youden’s index was 0.577, 0.577 and 0.651, respectively (Fig. 3).

Furthermore, the VFA measured by QCT and BIA was divided into two groups according to the health and visceral obesity. We found that the consistency of the two methods is moderate in unadjusted model, model 1 (Kappa = 0.532 and 0.536, Table 7) and good in model 2 (Kappa = 0.611, Table 7).

**Table 7:** The consistency of the VFA between BIA and QCT using the Kappa method according to health and visceral obesity.

Total (1263)	QCT-BIA (cm^2^)	Kappa	
Unadjusted model VFA (cm^2^)	< 100	≥ 100	
Health (< 101.9)	307	110	0.532
Visceral obesity (≥ 101.9)	159	687	
Model 1 VFA (cm^2^)	< 100	≥ 100	
Health (< 119.96)	406	234	0.536
Visceral obesity (≥ 119.96)	60	563	
Model 2 VFA (cm^2^)	< 100	≥ 100	
Health (< 118.83)	418	196	0.611
Visceral obesity (≥ 118.83)	48	601	

## Discussion

In the present study, high correlations among WC were found between BIA and anthropometry. In addition, the method of BIA to estimate VFA has a stronger correlation with VFA by QCT than BMI and WC in female and male subgroups. However, the consistency for VFA measured by BIA and QCT not perfect by ICC and Kappa analysis. The cut-off point of VFA measured by BIA was 118.83 cm^2^ and the consistency was good in model 2 (Kappa = 0.611) for female, implying when using BIA to assess whether a person is visceral obesity, we must take into consideration age, BMI and WC. In addition, to a certain extent, the BIA has still superiority for monitoring the VFA of the same person. To the best of our knowledge, the present study is the first to validate VFA measured by BIA against manual measurement and QCT in an adult Chinese population.

As we all known, BMI has been widely used in epidemiological studies and other settings to evaluate obesity. However, BMI cannot distinguish between subcutaneous fat and visceral fat. In comparison, a prior study revealed that WC is more reliable as a predictor of visceral fat in metabolic syndrome^20^. However, WC measured by manual measurements had higher inter- or intra-reader variability. In comparison, (semi-) automated measurements, such as BIA, have lesser variability, and have been widely used to measure body composition in recent years. In addition, a previous study found that BIA and manual methods for measuring WC are interchangeable^21^. In the present study, a high correlation between WC was found between BIA and manual measurement, which is very important, and there is the possibility for future research can focus on the use of BIA not only for WC but also other trunk circumferences.

Visceral adiposity has shown to be closely associated with increased risk of cardiovascular morbidity and mortality^2^. Some studies have investigated the underlying mechanisms by showing that the intra-abdominal fat could secrete a variety of adipocytokines and inflammatory factors, which may change energy storage, insulin sensitivity, low-grade inflammatory responses, and abnormal blood coagulation^22,23^, thereby leading to insulin resistance and metabolic syndrome, such as hypertension, diabetes, dyslipidemia and coronary heart disease. Since excessive visceral fat can lead to a series of pathophysiological changes, the accurate measurement of visceral fat is of critical importance to evaluate the adiposity and predict the risks of subsequent diseases. At present, the VFA could be accurately measured by QCT. A previous study has pointed to the umbilical plane or L2-L3 plane to evaluate the VFA^9,24^. In the present study, the QCT-VFA was measured with the L2-L3 plane. However CT scans are costly and time-consuming and expose patients to ionizing radiation. It is reported that multi-frequency BIA seems to be a more convenient, economic and nonradioactive method than QCT to estimate the VFA^25^. Accuracy of VFA estimated by BIA is affected by age, gender, race, exercise, disease state and so on and is estimated based on few factors (e.g. the impedance of the trunk, fat percentage, and muscle mass). In our study, the VFA-BIA was positively correlated with the VFA-QCT (r = 0.512); Furthermore, a higher consistency of VFA measurement was found in previous study (*r* = 0.920)^26^. In subgroups, the VFA estimated by BIA lost its consistency in male when compared with female (ICC = 0.623 for female and ICC = 0.174 for male). Contrary to our results, Lee et al. showed better consistency in male (ICC = 0.438) and female (ICC = 0.577)^27^, which may be different from race and life style. In addition, we found that the highest ICC value was 0.634 in female with age < 50 years, which corroborates the report^26^. In BMI subcategories, we showed the better consistency (ICC = 0.421 for BMI < 24 kg/m^2^ and ICC = 0.438 for BMI ≥ 24 kg/m^2^); Contrary to our results, Lee et al. showed better consistency in BMI < 25 kg/m^2^ (ICC = 0.496) compared with BMI ≥ 25 kg/m^2^ (ICC = 0.387), which was due to different BMI classification standards in different country. In addition to the higher consistency of female, the consistency of male and all is not most ideal. Furthermore, we found the moderate consistency between BIA and QCT in different subgroups according to gender and age by Kappa analysis, indicating that although the VFA measured by BIA is lower than QCT, the VFA measured by BIA was increasing with the increase of VFA by QCT to some extent.

To determine further whether the correlation of VFA and WC, BMI differed by manually, BIA and QCT, we focused in comparing the correlation according to gender. Our data showed that the correlation of the VFA between QCT and BIA estimated by BMI was lower than that of WC, which is consistent with a previous study^28^. This may indicate that WC is more closely correlated to visceral fat, when compared to BMI. However, another study suggested that the fat mass could be predicted by the combination of WC and BMI^29^. The investigators followed this suggestion, and combined WC and BMI to predict the VFA. It was observed that the method lost its accuracy in females. The present results of WC having a stronger consistency with VFA, when compared to BMI, are consistent with the results that WC is best correlated with VFA by CT in males and females^28^. When adding total body fat onto BMI, this becomes inadequate to predict the visceral adipose with the increment of BMI^30^. Furthermore, WC was a better descriptive and convenient method for these VFA variances, when compared to BMI. Our studies have also revealed that VFA estimated by BIA has a higher correlation with VFA by QCT than BMI and WC according to gender categories. Berker et al. demonstrated a best consistency between BIA and QCT in subgroups with BMIs < 25 kg/m^2^ and > 35 kg/m^2^, Consistent with our results, the Bland–Altman analysis showed BIA underestimated the higher VFA (> 100 cm^2^) and overestimated lower VFA (< 100 cm^2^) compared with the QCT, which was consistent with a VFA study measured between BIA and QCT^27^ and another study between BIA and DAX^31^. However we did not further analyze the BMI subgroup. This phenomenon may result in an underestimation of the percentage of body fat and an overestimation of fat free mass in morbid obesity.

It is noteworthy that the cut-off point of VFA measured by BIA was 118.83 cm^2^ in model 2 for female. The consistency in model 2 is better than unadjusted model and model 1 (Kappa = 0.611), implying that we must take into consideration gender, BMI and WC for assessing VFA by BIA. Ultimately, the VFA of the QCT can be predicted by age, BMI, WC and the VFA measured by BIA^26^. Whereas a more accurate formula is needed to match the CT data in China.

In addition, we found that males were observed to have higher overall adiposity and visceral fat, when compared to females, as demonstrated by the significantly higher values of VFA-QCT, BMI and WC (*P* < 0.05). This could suggest that females have given more attention to their body shape, while males neglect this a bit more probably due to higher pressure from life and work. Therefore, these present results highlight the importance of lifestyle intervention among males, in order to prevent obesity and related diseases.

This is likely the first study to validate VFA measured by BIA against manual measurement and QCT in China. The strength of the present study includes the measurements of comprehensive adiposity variables using different methods and the application of comprehensive statistical analyses, in order to examine the correlation into details. However, some limitations merit consideration. First, the present study was cross-sectional in nature. Zopfs, D et al. reported that the accurate prediction of patient visceral fat mass between BIA and QCT in 62 malignant melanoma^32^. In addition, Midori Ida et al. corroborated the significance of evaluating intra-abdominal fat area (IAFA) estimated by BIA as a biomarker for obesity compared with QCT during calorie restriction^33^, which indicates the usefulness of the BIA. Furthermore, the present study focused in comparing these different methods. Therefore, the cross-sectional study design was adequate for the aim. In addition, the study was conducted among a Chinese population.

In conclusion, our study has shown that VFA estimated by BIA was significantly correlated with VFA measured directly by QCT in female. In addition, the cut-off point of VFA measured by BIA was 118.83 cm^2^ in model 2 for female; in the light of the big LOA between the two methods by ICC and the good consistency in model 2 by kappa analysis, we must take into consideration gender, age, BMI and WC for assessing visceral obesity by BIA. In addition, WC is a more reliable predictor for intra-abdominal fat, when compared to BMI. Ultimately, in view of non-invasive and radiation-free of BIA, BIA can be used a way to measure anthropometric parameters in an easy and immediate manner, especially for monitoring the VFA of the same person. However, no uniform standard is available at present for measurements via BIA. Further studies are warranted to compare the clinical intervention results of the VFA and biochemical test of specimens in China, in order to obtain a more accurate formula is needed to match the QCT data and an standard for measuring VFA by BIA.

## References

[1] Xue H (2016). Incidence of type 2 diabetes and number of events attributable to abdominal obesity in china: A cohort study. J. Diabetes.

[2] Lavie CJ (2016). Obesity and prevalence of cardiovascular diseases and prognosis: The obesity paradox updated. Prog. Cardiovasc. Dis..

[3] Ahmad FS (2016). Hypertension, obesity, diabetes, and heart failure–free survival: The cardiovascular disease lifetime risk pooling project. JACC.

[4] Endocrinology Section of Chinese Medical Association (2011). Consensus of Chinese experts on adult obeisty. Chin. J. Endocrinol. Metab..

[5] Frayn KN (2000). Visceral fat and insulin resistance: Causative or correlative?. Br. J. Nutr..

[6] Levelt E (2016). Ectopic and visceral fat deposition in lean and obese patients with type 2 diabetes. J. Am. Coll. Cardiol..

[7] Miyawaki T (2005). Metabolic syndrome in Japanese diagnosed with visceral fat measurement by computed tomography. Proc. Jpn. Acad. B.

[8] Yoshizumi T (1999). Abdominal fat: Standardized technique for measurement at CT. Radiology.

[9] Kobayashi J, Tadokoro N, Watanabe M, Shinomiya M (2002). A novel method of measuring intra-abdominal fat volume using helical computed tomography. Int. J. Obes..

[10] He Q, Wang J, Engelson ES, Kotler DP (2003). Detection of segmental internal fat by bioelectrical impedance analysis in a biological phantom. Nutrition.

[11] Ryo M (2005). A new simple method for the measurement of visceral fat accumulation by bioelectrical impedance. Diabetes Care.

[12] Cha K (1997). Evaluation of segmental bioelectrical impedance analysis (SBIA) for measuring muscle distribution. J. Ichper Sd-Asia.

[13] Chen C, Lu FC (2004). The guidelines for prevention and control of overweight and obesity in Chinese adults. Biomed. Environ. Sci..

[14] Salinari S (2002). New bioimpedance model accurately predicts lower limb muscle volume: Validation by magnetic resonance imaging. Am. J. Physiol.-Endocrinol. Metab..

[15] Salinari S (2003). Bioimpedance analysis: A useful technique for assessing appendicular lean soft tissue mass and distribution. J. Appl. Physiol..

[16] Tanaka S (2019). The decreasing phase angles of the entire body and trunk during bioelectrical impedance analysis are related to locomotive syndrome. J. Orthop. Sci..

[17] Tanaka S (2018). Relationship between locomotive syndrome and body composition among community-dwelling middle-age and elderly individuals in Japan: The Yakumo study. Mod. Rheumatol..

[18] Ranganathan P, Pramesh CS, Aggarwal R (2017). Common pitfalls in statistical analysis: Measures of agreement. Perspect. Clin. Res..

[19] R Core Team. *R: A language and environment for statistical computing*. R Foundation for Statistical Computing, Vienna, Austria. https://www.R-project.org (2019).

[20] Mitsuhiro K, Takeshi Y (2012). Visceral fat area, waist circumference and metabolic risk factors in abdominally obese Chinese adults. Biomed. Environ. Sci..

[21] Tanaka S (2019). Waist circumference measured by bioelectrical impedance analysis is interchangeable with manual measurement: Increased waist circumference is associated with locomotive syndrome risk. Biomed. Res. Int..

[22] William T, Turner JE, Dylan T (2018). Parallels in immune metabolic adipose tissue dysfunction with ageing and obesity. Front. Immunol..

[23] Calabro P, Yeh ET (2007). Intra-abdominal adiposity, inflammation, and cardiovascular risk: New insight in the global cardiometabolic risk. Curr. Cardiovasc. Risk Rep..

[24] Han TS (1997). Relationship between volumes and areas from single transverse scans of intra-abdominal fat measured by magnetic resonance imaging. Int. J. Obes..

[25] Demura S, Sato S (2007). Prediction of visceral fat area at the umbilicus level using fat mass of the trunk: The validity of bioelectrical impedance analysis. J. Sports Sci..

[26] Nagai M (2008). Development of a new method for estimating visceral fat area with multi-frequency bioelectrical impedance. Tohoku J. Exp. Med..

[27] Lee DH (2015). Comparison of abdominal visceral adipose tissue area measured by computed tomography with that estimated by bioelectrical impedance analysis method in Korean subjects. Nutrients.

[28] Berker D (2010). Compatibility of different methods for the measurement of visceral fat in different body mass index strata. Diagn. Interv. Radiol..

[29] Berentzen TL (2012). Waist circumference adjusted for body mass index and intra-abdominal fat mass. PLoS ONE.

[30] Suh YS, Kim DH, Lee I (2002). Usefulness of lumbar AP spine DXA for measuring the percentage of perilumbar regional fat and predicting visceral fat in obese postmenopausal women. Nutrition.

[31] Demura S, Sato S (2007). Prediction of visceral fat area at the umbilicus level using fat mass of the trunk: The validity of bioelectrical impedance analysis. J. Sports.

[32] Zopfs D (2019). Single-slice CT measurements allow for accurate assessment of sarcopenia and body composition. Eur. Radiol..

[33] Ida M (2013). Early changes of abdominal adiposity detected with weekly dual bioelectrical impedance analysis during calorie restriction. Obesity.

